# Psychometric investigation of the French version of the Aberrant Salience Inventory (ASI): differentiating patients with psychosis, patients with other psychiatric diagnoses and non-clinical participants

**DOI:** 10.1186/s12991-020-00308-0

**Published:** 2020-10-03

**Authors:** Philippe Golay, Julien Laloyaux, Mihaela Moga, Clara Della Libera, Frank Larøi, Charles Bonsack

**Affiliations:** 1 Community Psychiatry Service, Department of Psychiatry, Lausanne University Hospital and University of Lausanne, Consultations de Chauderon, Lausanne, Switzerland; 2 General Psychiatry Service, Treatment and Early Intervention in Psychosis Program (TIPP–Lausanne), Lausanne University Hospital and University of Lausanne, Place Chauderon 18, 1003 Lausanne, Switzerland; 3 Institute of Psychology, Faculty of Social and Political Science, University of Lausanne, Lausanne, Switzerland; 4 Psychology and Neuroscience of Cognition Research Unit, University of Liège, Liège, Belgium; 5 Department of Biological and Medical Psychology, University of Bergen, Bergen, Norway; 6 NORMENT–Norwegian Center of Excellence for Mental Disorders Research, University of Oslo, Oslo, Norway

**Keywords:** Aberrant salience, Questionnaire, Psychotic proneness, Validity, Reliability, French validation

## Abstract

During the prodromal phase of psychosis, individuals may experience an aberrant attribution of salience to irrelevant stimuli. The concept of aberrant salience has been hypothesized to be a central mechanism in the emergence and maintenance of psychosis. The 29-item Aberrant Salience Inventory (ASI) was designed to measure five aspects of aberrant salience. The aim of this study was to investigate the psychometric properties of the French version of the ASI comparing patients with psychosis, patients with other diagnosis and healthy, non-clinical participants. The French-language ASI was adapted using the back-translation procedure. Two hundred and eighty-two participants issued from the general population and 150 psychiatric patients were evaluated. Internal validity was assessed using a two-parameter logistic item response model. Reliability was estimated using a test–retest procedure. Convergent validity was estimated using correlations between the ASI scores and several other scales. Sensitivity was evaluated by comparing the scores of participants with a diagnosis of psychosis, patients with other diagnoses and the general population. The best model distinguished three factors: Enhanced Interpretation and Emotionality, Sharpening of Senses and Heightened Cognition. Reliability and convergent validity estimates were good in both groups. The Sharpening of Senses factor was able to discriminate between patients and the general population. Only the Heightened Cognition factor was able to discriminate patients with psychosis from the other psychiatric patients. The ASI is a valid and reliable tool to study not only the aberrant salience phenomenon in patients with psychosis, but also with other diagnoses and within the general population.

## Introduction

Schizophrenia is a neurodevelopmental disorder affecting about 1% of the world population and reflecting a convergence of genetic risk factors and early life stress [9, 20]. The clinical characteristic of schizophrenia is psychosis, that includes experiences such as hallucinations (aberrant perceptions) and delusions (fixed, false beliefs) [6, 23]. Previous research has shown that psychosis emerges gradually, with a prodromal phase which varies in duration from several weeks to several years or longer [38]. During the prodromal phase of psychosis, individuals experience an aberrant attribution of salience to otherwise irrelevant stimuli. Indeed, the concept of aberrant salience has been hypothesized to be a central mechanism in the emergence of psychosis, and an important identifier of subjects at risk of developing the illness [6, 23, 24].

In an influential article entitled “*Psychosis as a state of aberrant salience*”, Kapur [23] proposed the aberrant salience hypothesis of psychosis. According to this hypothesis, dysfunctional mesolimbic dopamine release leads to an abnormal attribution of significance to external and internal stimuli. Thus, dopamine mediates “*the salience of environmental events and internal representations (…) Delusions are a cognitive effort by the patient to make sense of these aberrantly salient experiences, whereas hallucinations reflect a direct experience of the aberrant salience of internal representations*” [23]. Previous studies have found that dopaminergic anomalies may contribute to aberrant salience involving both rewarding and aversive signalling, which could generate feelings of apprehension and the impression that the world is changing [3, 18]. According to Jaspers [21], this state characterizes the prodromal phase preceding psychosis, referred to as a *delusional atmosphere.* Furthermore, some cognitive models of psychosis [15, 26] describe the factors that shape and maintain the positive psychotic symptoms: there is a biopsychosocial vulnerability that can be triggered by stressful or traumatic events, but the person’s appraisal of them plays a key role in symptom formation, such as persecutory delusions.

To our knowledge, there are two instruments that assess this concept of aberrant salience: the Salience Attribution Test (SAT; [35] and the Aberrant Salience Inventory (ASI; [6]. The SAT is a probabilistic computer-based learning game, rewarded with money, in which participants are required to quickly respond to task-relevant and task-irrelevant cue features. The test measures the adaptive (relevant) and aberrant (irrelevant) motivational salience [35], but the SAT can only be applied on small samples due to financial and time resource-constraints. The ASI is a valid, reliable, and easy to administer self-report questionnaire that measures the degree of aberrant salience [6]. Previous studies have found that the ASI is strongly correlated with psychosis-proneness symptoms such as magical ideation [11] or perceptual aberration [5]. The Italian [30] and the Spanish [13] versions of the ASI possess good psychometric qualities. However, an examination of the psychometric properties of a French version of the ASI is lacking. Thus, the main aim of this study was to assess the psychometric properties of the French version of the ASI in both clinical and non-clinical samples. Another objective was to identify the clinical cut-off score of the ASI.

## Material and methods

### Participants

A total of 432 French-speaking individuals participated in the study. The first sample was made up of 282 participants from the Belgian general population and was recruited online. The second sample consisted in 150 persons hospitalized in various psychiatric institutions in Switzerland.

The general population sample included 282 persons, 72% (*n* = 203) were students and 75% (*n* = 211) were female. Participants ranged from 18 to 58 years old, with a mean age of 23.85 (*SD* = 7.64). Roughly 53% (*n* = 149) were single or divorced and 47% (*n* = 133) were in a relationship or married. None of the participants reported having a current mental disorder. Roughly 86% (*n* = 242) of participants never had any mental problems in the past, whereas 16% (*n* = 40) had suffered from depression and/or anxiety disorders in the past. All participants provided informed consent and completed the online survey. To ensure data quality, 20 participants were excluded due to an extreme score (≥ 2.68 SD) on 6 validity items. The validity items consisted of two items aimed to detect random completion or attention lapses (e.g., “please answer XX for this question”), two items to detect a lie (issued from the Eysenck Personality Questionnaire Revised; [12] and two items were designed to detect the simulation of psychotic symptoms and are based on publicized clichés (issued from [33]. Eighteen additional participants were excluded because they reported a current psychiatric disorder, 1 because of current neuroleptic medication and 13 because they were consulting a mental health professional.

Participants from the clinical sample included 150 patients that were recruited during their hospitalization in different psychiatric hospitals or in other residential facilities from three French-speaking Swiss cantons (Fribourg, Vaud and Neuchâtel). They were approached by research assistants (trained master’s degree psychology students or 6th year medical students) in the presence of their attending nurse or doctor. Participants were informed about the study and those interested in participating were assessed individually after having given written consent. Mean age was 40.6 (SD = 12.81) years and 63% (*n* = 94) were male. Almost 73% (n = 109) of the participants were born in Switzerland, 83% (*n* = 124) had Swiss nationality and all of them were native or proficient French speakers. Only 12.7% (*n* = 19) of the participants were married, the rest were single, divorced, separated or widowed. Primary diagnostic categories based on the International Statistical Classification of Diseases and Related Health Problems 10th Revision (ICD-10) were: 50% (*n* = 75) Psychosis, 16.7% (*n* = 25) Depression, 12% (*n* = 18) Mania, 6.7% (*n* = 10) Personality disorder, 4.0% (*n* = 6) Anxiety and stress-related disorder and 6% (*n* = 9) other diagnoses.

### Measures

#### The French version of the Aberrant Salience Inventory (ASI)

The ASI is a self-report questionnaire that measures aberrant salience and psychosis proneness [6]. The 29 items have a dichotomous response format on a true–false scale. The original inventory has a single second-order factor and five first-order factors: *Increased Significance, Sharpening of Senses, Impeding Understanding, Heightened Emotionality and Heightened Cognition. Increased Significance* (items 1, 5, 10, 15, 16, 21 and 27) refers to the assignment of significance to otherwise innocuous stimuli. A typical item is: *“Do certain trivial things ever suddenly seem especially important or significant to you?”*. The *Sharpening of Senses* (items 3, 9, 12, 18 and 22) refers to anomalies of perceptions and subjective feelings of greater acuteness of the senses. An example of an item is: *“Do your senses sometimes seem sharpened?*”. *Impeding Understanding* (items 2, 6, 11, 17 and 29) refers to an increased sense of meaning and feelings of being close to a breakthrough in understanding. A typical item is *“Do you sometimes feel like you are on the verge of something really big, but you’re not sure what it is?”. Heightened Emotionality* (items 8, 14, 20, 24, 26 and 28) and *Heightened Cognition* (items 4, 7, 13, 19, 23 and 25) are related to emotions and cognitive abilities that accompany the attempt of finding an explanation to the aberrant salience experience. Typical items are, respectively, *“Do you ever have difficulty telling if you are thrilled, frightened, pained, or anxious?”* and “*Do you ever feel like you are rapidly approaching the height of your intellectual powers?”.*

The ASI was translated into French by Charles Bonsack, Julien Laloyaux, Philippe Golay and Imane Semlali, and then back translated into English by an independent professional translator. This translation was then examined by the authors of the original scale (i.e. David C. Cicero). No noteworthy changes were required upon examination of this translation.

#### The Highly Sensitive Person Scale (HSPS)

The HSPS is composed of 27 items and measures sensory-processing sensitivity, which involves high sensory sensitivity and associated arousability [1]. Participants rated how they generally feel on a 7-point Likert scale ranging from 1 (*not at all*) to 7 (*extremely*)*.* Typical items are: *“Are you easily overwhelmed by strong sensory input?”* or *“Do other people's moods affect you?”*. High scores reflect a high level of sensitivity. In our study, we used the French-version of the HSPS. The internal consistency of the HSPS in the current samples was good (general sample: *α* = 0.84; clinical sample: *α* = 0.88).

#### The Internal and External Encoding Style Questionnaire (ESQ)

The ESQ is a 21-item questionnaire designed to measure individual differences in how encoding is affected by information coming directly from the senses versus from preexisting schemata [31]. Participants rate, on a 6-point Likert scale, ranging from 1 (*strongly disagree*) to 6 (*strongly agree*), the frequency of having experiences of “split-second illusions”, that indicate the hasty application of the preexisting interpretative categories. Typical items are: “*Sometimes when I’m driving, I see a piece of paper or a leaf being moved by the wind and for a split second think it might be an animal (e.g., a squirrel or a cat)”* or *“I’ve sometimes noticed a particular object to my left or right, and only after I turned my head I realized it was something else”.* There are only six diagnostic items (5, 8, 11, 15, 18 and 21); the 15 other items are included in order to conceal the focus of the questionnaire. Lewicki [31] assumed that the two encoding styles range on a continuum from “extremely internal” to “extremely external”. A high score on the ESQ indicates an internal encoding style, whereas a low score reflects an external encoding style. In our study, we used the French version of the ESQ [2] and its internal consistency was satisfactory in the clinical sample (*α* = 0.79). As the scale consisted of only six diagnostic items, its internal consistency in the general sample can be considered as being adequate (*α* = 0.66).

#### The Magical Ideation Scale (MIS)

The MIS is a 30-item true/false questionnaire measuring *“belief in forms of causation that by conventional standards are invalid”* and is considered a general measure of schizophrenia proneness [11]. Typical items include superstitions, magical beliefs, and the capacity to read one’s thoughts (e.g., “*Numbers like 13 and 7 have no special powers”,* or “*I have sometimes felt that strangers were reading my mind”*). There are 7 reverse-scored items (4, 7, 15, 19, 22, 24 and 30) and 23 standard items. The total score ranges from 0 to 30, with high scores reflecting high levels of magical thinking. In the present study, we used the French version of the MIS [10] and its internal consistency was good in both samples (general sample: *α* = 0.80; clinical sample: *α* = 0.86).

#### The Perceptual Aberration Scale (PAS)

The PAS is a 35-item true/false inventory measuring psychotic-like perceptual distortions [5]. Typical items describe perceptions of one’s own body (e.g., *“I sometimes have had the feeling that my body is abnormal”*) or other perceptual distortions (e.g., *“My hearing is sometimes so sensitive that ordinary sounds become uncomfortable”*). High scores reflect high levels of perceptual aberration. In our study, we used the French version of the PAS [10] and its internal consistency was good in the general sample (*α* = 0.87) and excellent in the clinical sample (*α* = 0.90).

#### The Launay–Slade Hallucinations Scale (LSHS)

The LSHS is a widely used questionnaire designed to measure hallucinatory experiences [27, 28]. The original English version of the LSHS consisted of 12 items answered on a true/false response format [29]. The revisited version used in our study had 17 items answered on a 5-point Likert scale ranging from 0 (*certainly does not apply to me*) to 4 (*certainly applies to me*). The total score ranges from 0 to 68, with high scores reflecting a greater predisposition towards hallucinations. This scale was only administered to the clinical sample and its internal consistency was excellent (*α* = 0.90).

### Procedure

In order to assess the internal validity of the French-language ASI scores, we tested the original five-factor ASI model. Given the pattern of results, we also tested a three- and a one-factor alternative. In order to test the reliability of the ASI scores, we used a test–retest aproach with an interval ranging from 2 to 14 days. The retest questionnaire was completed by 40 participants from the clinical sample. We also computed internal consistency estimates based on the first assessment. In order to estimate the convergent validity, we examined the relationship between ASI scores and scores from several other scales. We hypothesized that ASI scores would be positively correlated with the Highly Sensitive Person Scale (HSPS), the Encoding Style Questionnaire (ESQ), the Magical Ideation Scale (MIS), the Perceptual Aberration Scale (PAS) and the Launay–Slade Hallucinations Scale (LSHS).

Finally, we assessed the sensitivity of the ASI based on the hypothesis that the participants suffering from a psychotic disorder can be discriminated from other populations based on higher ASI scores.

### Statistical analysis

#### Internal validity

Due to the items’ dichotomous nature, internal validity was estimated using two-parameter logistic (2PL) item response models. The models were estimated using a robust weighted least squares estimator with adjustments for the mean and variance (WLSMV). These estimator models were compared with a robust Chi-square test using the DIFFTEST procedure. Several indicators of model fit were used: the Root Mean Square Error of Approximation (RMSEA), the Comparison Fit Index (CFI) and the Tucker–Lewis fit Index (TLI). RMSEA values ≤ 0.06, and CFI and TLI values ≥ 0.95, were interpreted as good fits, whereas RMSEA values ≤ 0.08, and CFI and TLI values ≥ 0.90 were considered as indicating satisfactory fit [19].

#### Reliability

The reliability of the ASI subscales was estimated using McDonald’s model-based Omega (*ω*) [4] and Cronbach’s alpha (*α*) coefficients. The test–retest reliabilities were estimated using both Pearson and intraclass correlation coefficients using a two-way random-effects model and the absolute agreement definition (ICC [2, 1]). Reliability coefficients above 0.70 were considered satisfactory; above 0.80 were considered good; and above 0.90 were considered excellent [4, 16].

#### Convergent validity

The convergent validity coefficients between the ASI subscales and the other scales were estimated using Pearson correlation coefficients. Under Classical Test Theory (CTT) the score reliabilities (more precisely their square root) act as an upper bound for validity coefficients. Therefore, the acceptable range is typically lower than for reliability coefficients. Correlation coefficients between 0.40 and 0.60 were considered as good and any values higher than 0.30 (a medium effect size, according to Cohen [7] as satisfactory.

#### Sensitivity

The sensitivity of the French version of the ASI was examined by comparing three groups: participants with a diagnosis of psychosis, people with another psychiatric diagnosis, and the general population without a psychiatric diagnosis. The three groups were compared using a Bayesian approach which represents an elegant alternative to the classic problem of multiple comparisons [17]. All five possible Gaussian (*μ*, *σ*^2^) models were estimated. The first model was the homogeneous model (scores from the three groups are issued from the same distribution). This model was referred as “(1, 2, 3)” and corresponded to the null hypothesis in the classical statistical testing framework. Another model was the heterogeneous model “(1) (2) (3)” that states that the scores from the three groups differ from each other and are issued from three different distributions. The three models “(1) (2, 3)”, “(1, 2) (3)” and “(1, 3) (2)” were also estimated and indicate than one of the three groups differ from the two other groups. The best model was determined by using the BIC (Bayesian Information Criterion) [36]. The BIC coefficients were used to calculate the Bayes factor and the posterior probability [25]. The Bayes factor provided a comparison of the best model with the homogenous model. A Bayes factor of 4 would indicate that the best model is four times more likely to be true than the homogenous model. Values over 3 are generally considered as sufficiently important to favour one model over another [22, 37]. An equal prior probability of 1/5 was assumed for all models.

Finally, when a score was able to discriminate between groups, a receiver operating characteristic (ROC) curve was estimated in order to identify the area under the curve, the sensitivity, the specificity and an ideal cut-off score between the groups. The cut-off score was fixed in order to maximize the product of the sensitivity and specificity. All statistical analyses were performed with IBM-SPSS 25 and the AtelieR package for R [34].

## Results

### Internal validity

**Fig. 1:**
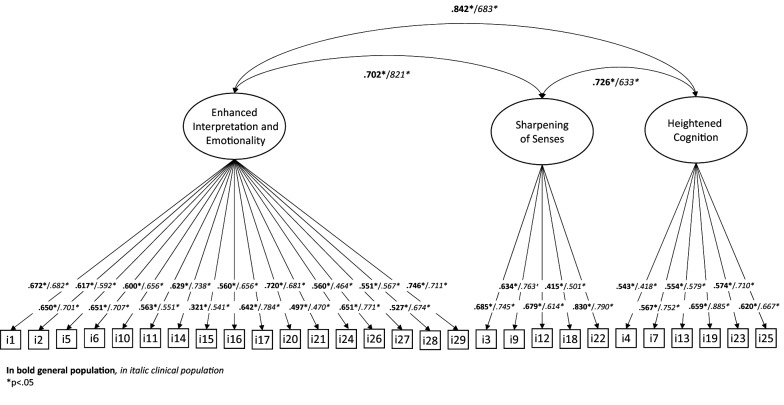
Factor loadings of the ASI

The model fit of the original five factors model was good in the general population sample and in the clinical sample (Table 1). However, several correlations between the five factors were very high both in the clinical sample (3 correlations > 0.90) and in the general sample (4 correlations > 0.90). Additionally, the loading of item 8 did not reach statistical significance in the clinical group. We therefore decided to exclude item 8 and to estimate a simpler three-factor model by collapsing factors with very high correlations in one unique “Enhanced Interpretation and Emotionality” factor (Fig. 1). The model fit was excellent in both groups and all factor loadings were significant. For the sake of parsimony, we tested an additional, simpler one-factor model. While its fit was good, the direct comparison with the three-factor model revealed that the three-factor structure was the preferred solution in both samples.

**Table 1:** Comparisons of model fit for the ASI

Model	*χ*^2^	df	*p*-value	RMSEA	90% C.I. for RMSEA	CFI	TLI	Remarks
General population (*N* = 282)								
(a) Original five-factor model	511.981	367	< 0.001	0.037	0.029–0.045	0.938	0.932	4-factor correlations > 0.90
(b) Three-factor model^a^	500.910	347	< 0.001	0.040	0.032–0.047	0.932	0.926	−
(c) One-factor model^a^	548.720	350	< 0.001	0.045	0.038 –0.052	0.912	0.905	−
(b) vs (c)								(b) better than (c) (Δχ^2^ = 41.695, Δdf = 3, p < .001)
Clinical population (*N* = 150)								
(a) Original five-factor model	437.586	367	.007	0.036	0.020–0.048	0.955	0.950	3-factor correlations > 0.90 and loading of item 8 not significant
(b) Three-factor model^a^	403.218	347	.020	0.033	0.014 –0.046	0.964	0.961	−
(c) One-factor model^a^	451.269	350	< 0.001	0.044	0.031–0.055	0.935	0.930	−
(b) vs (c)								(b) better than (c) (Δ*χ*^2^ = 38.963, Δdf = 3, p < 0.001)

df, degrees of freedom; RMSEA, Root Mean Square Error of Approximation; CI, confidence Interval; CFI, Comparative Fit Index; TLI, Tucker–Lewis Index; ^a^ Item 8 excluded

### Reliability

Internal consistency Omega estimates were good for all three scores in both groups (Table 2). Cronbach alphas were typically lower for the Sharpening of Senses and the Heightened Cognition scores. Test–retest reliability was satisfactory except for the Sharpening of Senses score which was relatively poor.

**Table 2:** Reliability of the ASI scores

	Internal consistency		Test–retest reliability (*N* = 40)	
	McDonald’s *ω*	Cronbach’s *α*	Pearson’s r	ICC (2,1)
General population (*N* = 282)				
Enhanced interpretation and Emotionality	0.911	0.818	−	−
Sharpening of senses	0.802	0.604	−	−
Heightened cognition	0.802	0.550	−	−
Clinical population (*N* = 150)				
Enhanced interpretation and Emotionality	0.930	0.849	0.701*	0.700*
Sharpening of senses	0.840	0.667	0.574*	0.527*
Heightened cognition	0.848	0.679	0.718	0.710*

*  = *p* < 0.05

### Convergent validity

The three ASI scores were significantly and positively correlated with all scales in both the general population and in the clinical group (Table 3).

**Table 3:** Convergent validity of the ASI scores

	ASI		
	Enhanced interpretation and emotionality	Sharpening of senses	Heightened cognition
General population (*N* = 282)			
Highly Sensitive Person Scale (HSPS)	0.401*	0.254*	0.306*
Internal and External Encoding Style Questionnaire (ESQ)	0.415*	0.176*	0.251*
Magical Ideation Scale (MIS)	0.601*	0.410*	0.534*
Perceptual Aberration Scale (PAS)	0.547*	0.353*	0.453*
Launay–Slade Hallucinations Scale (LSHS)	N/A	N/A	N/A
Clinical population (*N* = 150)			
Highly Sensitive Person Scale (HSPS)	0.513*	0.396*	0.198*
Internal and External Encoding Style Questionnaire (ESQ)	0.488*	0.450*	0.321*
Magical Ideation Scale (MIS)	0.576*	0.396*	0.673*
Perceptual Aberration Scale (PAS)	0.559*	0.444*	0.342*
Launay–Slade Hallucinations Scale (LSHS)	0.584*	0.448*	0.460*

* *p* < 0 .05; N/A = not available in the general population group

### Sensitivity

The Enhanced Interpretation and Emotionality score did not discriminate between patients with psychosis, other patients, and the general population (Table 4). The Sharpening of Senses score was able to discriminate between psychiatric patients and the general population but not between patients with or without psychosis. The Heightened Cognition score was able to discriminate between patients with psychosis and the other two groups (other psychiatric patients and the general population).

**Table 4:** Comparisons between the general population, patients with other diagnoses and patients with psychosis

	(1) General population *N* = 282 Mean (SD)	Psychiatric patients		Best model^a^	Bayes Factor against null hypothesis^b^	Probability of the model to be true^c^
		(2) Clinical population *N* = 71 Mean (SD)	(3) Psychosis *N* = 79 Mean (SD)			
Enhanced Interpretation and Emotionality	9.03 (3.97)	9.75 (4.20)	10.24 (4.60)	(1,2,3)	1.00	0.438
Sharpening of Senses	1.85 (1.48)	2.79 (1.53)	2.94 (1.65)	(1), (2,3)	5.38 * 10^7^	0.946
Heightened Cognition	1.80 (1.47)	2.11 (1.81)	2.95 (1.76)	(1,2), (3)	1.14 * 10^5^	0.861

^a^On the basis of the BIC coefficient

^b^Bayes factor comparing the best model with the homogeneous model (1, 2, 3)

^c^Among all possible models ((1, 2, 3) / (1, 2) (3) / (1) (2, 3) / (1, 3) (2) / (1) (2) (3))

ROC curves comparing the general population versus psychiatric patients for the Sharpening of Senses score yielded an area under the curve of 0.678, a sensitivity of 0.627, a specificity 0.667 and an optimal cut-off score of 2.5. ROC curves comparing patients with psychosis versus the general population and the other psychiatric patients for the Heightened Cognition score yielded an area under the curve of 0.676, a sensitivity of 0.582, a specificity of 0.703 and an optimal cut-off score of 2.5.

## Discussion

The best model distinguished three factors: Enhanced Interpretation and Emotionality, Sharpening of Senses and Heightened Cognition. While the hypothesized five-factor structure showed a good fit, several factor correlations were very high and thus the three-factor model was preferable in both the general and the clinical population. High factor correlations were also reported in the original study [6], but to a lesser extent. While these differences could tentatively be explained by characteristics of the samples (for instance difference in illness severity), they can also stem from methodological differences alone. In the original study, standard errors were treated with a robust estimator, but the dichotomous nature of items was not taken into account. In our study, we relied upon an item response model which precisely does that. The more traditional linear factor analytic model can underestimate correlations and shared variance between binary items. Therefore, this could explain why factors appeared more distinct in Cicero et al. [6].

The reliability estimates were good, except for the Cronbach Alphas of scores with the lowest number of items, which was to be expected. Indeed, the Alpha coefficient is notoriously biased when the number of items is low. Furthermore, it assumes unidimensionality, tau-equivalence (same factor loadings) and no residual correlations. Therefore, McDonald’s model-based Omega estimates provide more reliable information about reliability. Taken altogether, reliability estimates of the French translation of the ASI revealed could be considered as good.

Significant correlations with other scales suggested good convergent validity for all scores in both groups. In other words, the hypothesized relationships could also be observed in a non-clinical population which gives support to the adequateness of the aberrant salience concept within the general population.

Sensitivity was poor for the Enhanced Interpretation and Emotionality score which did not distinguish between patients and the general population nor between patients with psychosis and other psychiatric patients. However, this score was significantly related to other scales in both groups, suggesting inter-individual differences are not random and that there may be meaningful inter-individual differences. However, given that the average score was not different between groups, this factor cannot be used for diagnostic purposes.

Sharpening of Senses was able to discriminate patients from the general population, but not patients with psychosis. This suggests that some aspects of aberrant salience may not be psychosis-specific and this warrants further investigation. Finally, the Heightened Cognition score was able to distinguish patients with psychosis from other patients. This suggests that this dimension from aberrant salience might be the most specific to psychosis. This relationship between psychotic symptoms (unusual thought content) and Heightened Cognition has been found in a recent paper [8]. A network hypothesis involving the insula, the fronto-insular operculum, and the dorsal anterior cingulate cortex was proposed to account for the specificity of this domain for patients with psychosis.

Cut-off scores were suggested on the basis of our data, but sensitivity and specificity were far from excellent. At this stage, it remains difficult to decide whether this is due to a measurement issue with the ASI or due to a theoretical issue with the aberrant salience concept.

Our study has several limitations that could be the focus of future studies. First, despite extensive convergent validity estimation, prediction of conversion to psychosis was not investigated. The onset of psychosis is preceded by a prodromal phase of subclinical symptoms that represents both a period of vulnerability and an opportunity to early intervention [9, 32]. Since the earliest possible detection and intervention could improve outcomes for people at risk for developing a psychosis, there is a growing interest in the early identification, diagnosis and treatment for psychosis [14]. Aberrant salience could represent a key concept for identifying people at risk of developing a psychosis. Second, aspects of aberrant salience that may not be psychosis-specific should be studied in other, larger samples. This also warrants more theoretical scrutiny since this concept was born as an aberrant salience hypothesis of psychosis. Thirdly, some demographic characteristics differed between our general population and our clinical population samples. Patients were older and more likely to be men. Fourthly, while the same structural model was adequate in both samples, cut-off scores remain to be replicated in other samples. Finally, there is a need for exploring the measurement invariance between the clinical population and the healthy general population sample. In fact, while our pattern of result suggests configural invariance, metric and scalar invariances remains to be further studied.

## Conclusions

The ASI is a valid and reliable tool to study not only aberrant salience in patients with psychosis, but also with patients with other psychiatric diagnoses and in the general population.

## Appendix 1 French language version of the ASI

**Table:**

Questionnaire ASI [Cicero et al. 6]		
Instructions : Nous nous intéressons ici à l’étude des attitudes et expériences de vie que les gens ont. Le questionnaire qui suit contient de questions à propos de vos attitudes et expériences de vie. S’il vous plait, répondez par « Oui » ou « Non » après chaque question. En réfléchissant sur vous-même et à vos expériences, ne prenez pas en compte vos attitudes, sentiments ou expériences que vous auriez pu avoir sous l’influence d’alcool ou d’autres drogues (p.ex. cannabis, LSD, cocaïne).		
1. Arrive-t-il que certaines choses insignifiantes semblent soudainement particulièrement importantes ou significatives pour vous ?	Oui	Non
2. Avez-vous parfois l'impression que quelque chose de particulièrement important pour vous est sur le point d’arriver, mais sans être sûr de ce que c’est ?	Oui	Non
3. Vos sens semblent-ils parfois aiguisés ?	Oui	Non
4. Vous arrive-t-il de vous sentir comme si vous étiez en train d’atteindre rapidement le summum de vos facultés intellectuelles ?	Oui	Non
5. Remarquez-vous parfois des petits détails qui vous semblent importants alors que vous ne les aviez pas remarqués avant ?	Oui	Non
6. Vous sentez-vous parfois comme s’il était important pour vous de comprendre quelque chose, mais sans être sûr de ce que c’est ?	Oui	Non
7. Vous arrive-t-il de vivre des périodes durant lesquelles vous vous sentez particulièrement religieux ou mystique ?	Oui	Non
8. Vous arrive-t-il d’avoir des difficultés à dire si vous êtes ravi, effrayé, peiné ou anxieux ?	Oui	Non
9. Vous arrive-t-il de vivre des périodes de conscience accrue ?	Oui	Non
10. Vous arrive-t-il d’éprouver le besoin de donner un sens à des situations ou évènements qui semblent aléatoires ?	Oui	Non
11. Vous arrive-t-il d’avoir le sentiment d’être en train de trouver la pièce manquante d’un puzzle ?	Oui	Non
12. Avez-vous parfois le sentiment de pouvoir entendre avec une plus grande clarté ?	Oui	Non
13. Vous arrive-t-il de vous sentir comme si vous étiez une personne particulièrement évoluée au niveau spirituel ?	Oui	Non
14. Des observations habituellement insignifiantes prennent-elles parfois une signification de mauvaise augure ?	Oui	Non
15. Vous arrive-t-il de vivre des périodes pendant lesquelles les chansons semblent avoir un sens particulier pour votre vie ?	Oui	Non
16. Vous arrive-t-il parfois d’attribuer de l’importance à des objets auxquels vous n’en accorderiez normalement pas ?	Oui	Non
17. Avez-vous parfois l’impression d’être sur le point de comprendre quelque chose de vraiment grand ou d’important sans être sûr de ce que c’est ?	Oui	Non
18. Votre sens du goût vous a-t-il déjà semblé plus fin ?	Oui	Non
19. Vous arrive-t-il d’avoir l’impression que les mystères de l’univers se révélaient d’eux-mêmes à vous ?	Oui	Non
20. Vous arrive-t-il de vivre des périodes durant lesquelles vous vous sentez surstimulé par des choses ou des expériences qui sont habituellement gérables ?	Oui	Non
21. Vous arrive-t-il souvent d’être fasciné par les petites choses autour de vous ?	Oui	Non
22. Vos sens vous paraissent-ils parfois extrêmement forts ou clairs ?	Oui	Non
23. Vous arrive-t-il d’avoir l’impression qu’un monde entier s’ouvre à vous ?	Oui	Non
24. Vous arrive-t-il d’avoir l’impression que les frontières entre vos sensations internes et externes ont été enlevées ?	Oui	Non
25. Avez-vous parfois le sentiment que le monde est en train de changer et que vous cherchez une explication ?	Oui	Non
26. Vous arrive-t-il d’avoir un sentiment d’urgence inexprimable sans être sûr de ce qu’il faut faire ?	Oui	Non
27. Vous arrive-t-il parfois d'être intéressé par des personnes, des événements, des lieux, ou des idées qui ne devraient normalement pas retenir votre attention ?	Oui	Non
28. Arrive-t-il que vos pensées et vos perceptions surgissent plus vite qu’elles ne peuvent être assimilées ?	Oui	Non
29. Vous arrive-t-il de remarquer des choses que vous n’aviez pas remarquées avant et qui prennent une signification particulière ?	Oui	Non

”*Importance et émotionalité accrues*”= item 1 + item 2 + item 5 + item 6 + item 10 + item 11 + item 14 + item 15 + item 16 + item 17 + item 20 + item 21 + item 24 + item 26 + item 27 + item 28 + item 29; “*Aiguisement des sens*”= item 3 + item 9 + item 12 + item 18 + item 22; “*Cognition augmentée*”=item 4 + item 7 + item 13 + item 19 + item 23 + item 25.
